# Metal–Organic Frameworks as Formose Reaction Catalysts with Enhanced Selectivity

**DOI:** 10.3390/molecules28166095

**Published:** 2023-08-17

**Authors:** Valentina Balloi, Manuel Antonio Diaz-Perez, Mayra Anabel Lara-Angulo, David Villalgordo-Hernández, Javier Narciso, Enrique V. Ramos-Fernandez, Juan Carlos Serrano-Ruiz

**Affiliations:** 1 Materials and Sustainability Group, Department of Engineering, Universidad Loyola Andalucía, Avenida de las Universidades, s/n, 41704 Sevilla, Spain; vballoi@uloyola.es (V.B.); madiaz@uloyola.es (M.A.D.-P.); malangulo@uloyola.es (M.A.L.-A.); 2 Laboratory of Advanced Materials, Inorganic Chemistry Department, University Materials Institute of Alicante, University of Alicante, Apartado 99, 03080 Alicante, Spain; david.villalgordo@ua.es (D.V.-H.); narciso@ua.es (J.N.); enrique.ramos@ua.es (E.V.R.-F.)

**Keywords:** formose reaction, selectivity, metal–organic frameworks, heterogeneous catalysis, monosaccharides

## Abstract

The formose reaction is an autocatalytic series of aldol condensations that allows one to obtain monosaccharides from formaldehyde. The formose reaction suffers from a lack of selectivity, which hinders practical applications at the industrial level. Over the years, many attempts have been made to overcome this selectivity issue, with modest results. Heterogeneous porous catalysts with acid–base properties, such as Metal–Organic Frameworks (MOFs), can offer advantages compared to homogeneous strong bases (e.g., calcium hydroxide) for increasing the selectivity of this important reaction. For the very first time, four different Zeolite Imidazolate Frameworks are presented in this work as catalysts for the formose reaction in liquid phase, and their catalytic performances were compared with those of the typical homogeneous catalyst (i.e., calcium hydroxide). The heterogeneous nature of the catalysis, the possible contribution of leached metal or linkers to the solution, and the stability of the materials were investigated. The porous structure of these solids and their mild basicity make them suitable for obtaining enhanced selectivity at 30% formaldehyde conversion. Most of the MOFs tested showed low structural stability under reaction conditions, thereby indicating the need to search for new MOF families with higher robustness. However, this important result opens the path for future research on porous heterogeneous basic catalysts for the formose reaction.

## 1. Introduction

The formose reaction (FR) is the base-catalyzed oligomerization of formaldehyde (FA) to obtain monosaccharides. What we call FR is not just a simple reaction path but a series of mechanisms that include aldol condensation, retro aldol cleavages, equilibriums, and rearrangements. Moreover, other base-catalyzed reactions, such as the Cannizzaro and cross-Cannizzaro disproportions and the Lobry de Bruyn–van Ekenstein reaction, can compete with the main mechanism and proceed in parallel [1]. FR has been known since 1861 [2], and it has been widely studied. However, proper control of product selectivity still remains a challenge, mainly due to the fast and uncontrolled reactivity under the operating conditions studied up to date [3,4]. In fact, FR produces a complex mixture of linear and branched monosaccharides, polyols, and sugar acids, which makes this reaction unsuitable for any realistic industrial application. The majority of research in the past has focused on understanding its complex mechanism and on tuning its selectivity towards the formation of selected products [5,6,7,8,9,10,11,12,13,14,15,16]. In recent years, studies on the FR have primarily focused on its relevance concerning the origin of life. Some theories have hypothesized that sugars might have been formed in the prebiotic earth thanks to the highly alkaline environment, high temperatures, and the presence of minerals on the surface of the meteorites, along with the availability of FA [17,18,19,20,21,22,23,24,25,26,27].

An intense study in this field is being carried out, but few recent publications have addressed the issue of reaching an enhanced selectivity [28,29,30,31,32]. Homogeneous catalysts such as Ca(OH)_2_ [4,33] Ba(OH)_2_, Mg(OH)_2_, and Sr(OH)_2_ [7,14] have been widely used in this reaction because of their high activity in the aldol-condensation reaction. This high activity, however, is normally achieved at the expense of a significant lack of selectivity during the FR. The use of soluble thiazolium salts in the replacement of hydroxides has resulted in some control of the selectivity during the process [34,35]. Heterogeneous catalyst, such as natural minerals [36,37], zeolites [38,39], and oxides [40], have been reported to catalyze the FR, although with limited success in terms of control over the product distribution. Borate minerals and sodium silicate, with the ability to selectively coordinate certain sugars via complexation, have achieved control over the formose synthesis [41,42], although the results were not reproducible [43].

Metal organic frameworks (MOFs) are versatile and promising materials with applications in several areas, such as sensing [44], separation and sequestration [45], optics [46], electronics [47,48,49], molecular transport [50], and catalysis [51,52,53,54]. MOFs have been shown to efficiently and selectively catalyze organic reactions such as aldol condensation [55,56,57,58,59,60,61] and the Knoevenagel reaction [62]. Since the FR proceeds through subsequent aldol reactions, it is worth considering that MOFs could be an unexplored and valuable catalyst for the FR. Due to their porous structure and the numerous possibilities they offer in terms of ligand exchange [63], MOFs are promising materials for use as heterogeneous catalysts and for applications in challenging reactions such as FR. In this work, the FR catalyzed heterogeneously by different MOFs is explored for the first time. We used four different MOFs as catalysts for the FR: ZIF-67, 1,2,4-triazole exchanged ZIF-67 with 1:1 and 1:2 ratio, and a purchased ZIF-8. Results showed that it is possible to control the formose selectivity as compared to the traditional FR catalysts Ca(OH)_2_, generating a simple mixture of products and carrying out the reaction in a more controlled fashion.

## 2. Results and Discussion

ZIF-67 was prepared by mixing two water solutions of 2-methylimidazol and Co (NO_3_)_2_ with a high excess of ligand (metal to ligand ratio of 1:12). The solution was shaken for 2 minutes and subsequently left to stand for 6 h. The as-obtained solid was separated from the solution by centrifugation, washed with methanol several times, and centrifuged again. The as-obtained purple solid was finally dried at 60 °C. The exchanged samples were prepared by exchanging the ligand 2-methylimidazol with 1,2,4-1H-triazol. With this aim, methanolic solutions of ZIF-67 and the triazol ligand were mixed at two different ZIF-67 to triazol molar ratios (1:1 and 1:2, denoted as ZIF-67 exchanged 1:1 and ZIF-67 exchanged 1:2, respectively) under stirring for three days. The as-obtained exchanged MOF samples were separated from the solution by centrifugation and subsequently dried at 60 °C. ZIF-8 was commercially provided by Sigma-Aldrich.

**Figure 1:**
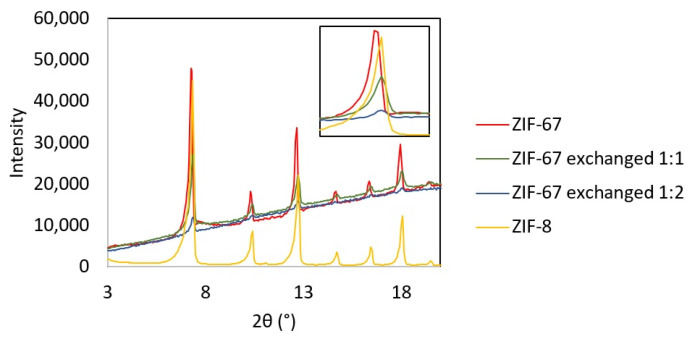
XRD patterns of MOFs samples. The zoom on the peak at 2θ 7.3° highlights the contraction suffered by the structure for the ligand substitution.

The synthesized ZIF-67, with different proportions of triazole exchange, and the purchased ZIF-8 were characterized by X-ray diffraction (XRD), and the diffraction patterns of all samples are reported in Figure 1. Comparing the patterns of ZIF-67, ZIF-8, and the exchanged samples, no difference in the structure is observed, confirming that the exchange process does not alter the framework. A slight shift towards higher angles is observed when analyzing the first peak at 2θ 7.3 for the exchanged samples, indicating a reduction in the d-spacing, i.e., a contraction of the structure with respect to unexchanged ZIF-67.

**Figure 2:**
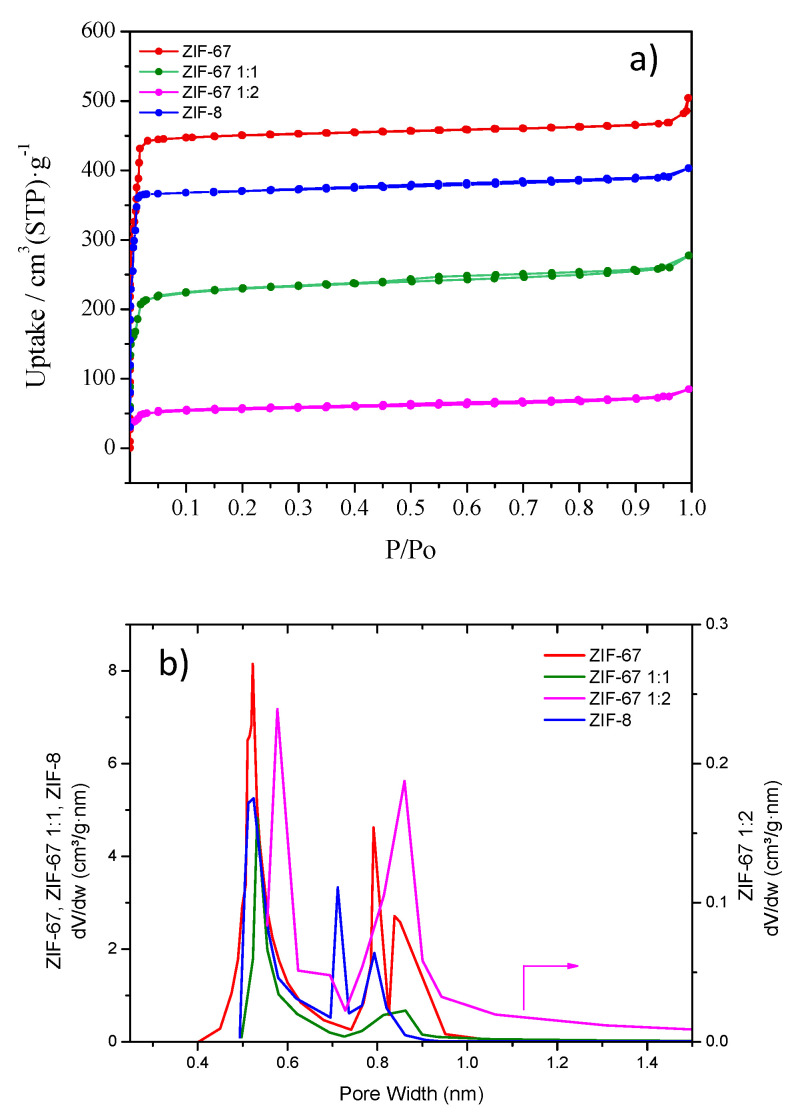
Nitrogen adsorption isotherms (**a**) and pore size distribution (**b**) of the four MOF samples under study.

The porosity of the MOF materials was studied by performing N_2_ adsorption measurements at 77 K (Figure 2a). The four MOF materials showed adsorption isotherms typical of pure microporous solids (i.e., type I). ZIF-67 (BET surface area: 1438 m^2^/g) and ZIF-8 (BET surface area: 1065 m^2^/g) showed larger surface areas and adsorption capacities than the exchanged samples ZIF-67 1:1 (BET surface area: 673 m^2^/g) and ZIF67 1:2 (BET surface area: 175 m^2^/g). The pore size analysis (Figure 2b) revealed a bimodal distribution for all the MOFs under study, with micropores of two sizes (0.5–0.6 nm and 0.7–0.9 nm). Interestingly, apart from a significant reduction in the surface area available, the ligand exchange process seemed to result in a slight widening of the micropores of lower size of the ZIF-67, which is particularly noticeable for the ZIF67 1:2 sample.

**Figure 3:**
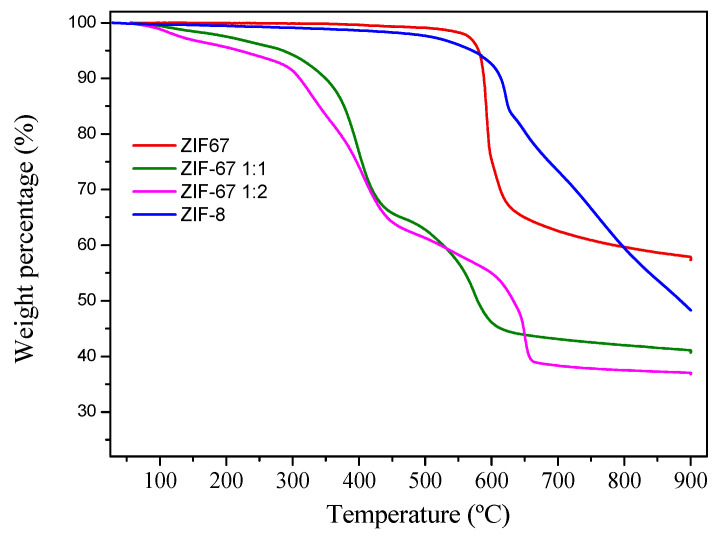
Thermogravimetric analysis for the four MOF samples under study.

The thermal stability of the MOF materials was studied by thermogravimetric analysis (TGA) under inert atmosphere (Figure 3). The ZIF-67 sample showed a single and abrupt weight loss episode at ca. 600 °C. The ligand exchange process altered the weight loss profile of ZIF-67. Thus, the exchanged samples ZIF-67 1:1 and ZIF-67 1:2 showed lower thermal stability than the parent material, showing two weight losses at 400 and 600 °C and generating less carbonized material after the experiments. The ZIF-8 sample showed a weight loss profile with a single step at ca. 650 °C, with slow and continuous decomposition until the end of the experiment.

**Figure 4:**
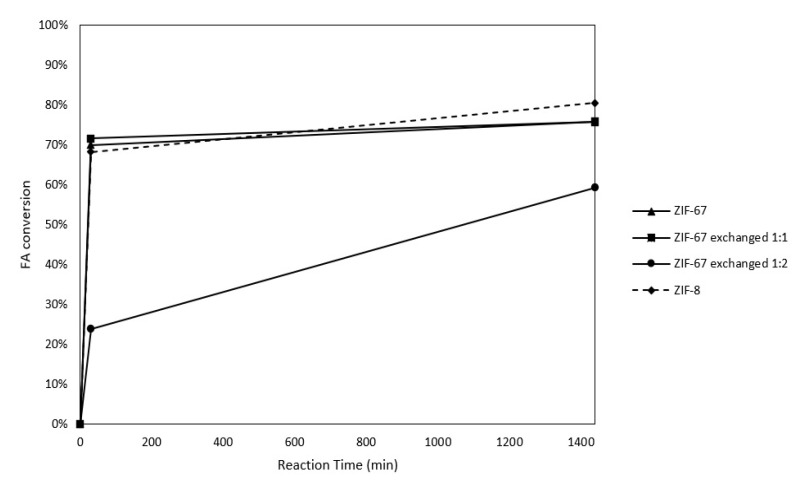
Conversion of FA as a function of time for an FR carried out at 60 °C, using different MOFs as catalysts.

To demonstrate the role of MOFs as catalysts of the FR, the materials were tested under the reaction conditions previously described. Figure 4 shows the conversion of FA as a function of the reaction time for each catalyst. Data are obtained from FA quantification by HPLC, as described previously. For ZIF-67, ZIF-67 exchanged 1:1 and ZIF-8, at 30 min of reaction time, the conversion is already around 70%; after this time, the conversion reaches a plateau and does not vary considerably. Interestingly, ZIF-67 exchanged 1:2 behaves differently, exhibiting a slower reaction, with only 30% of conversion at 30 min and less than 60% at 24 h of reaction time.

**Figure 5:**
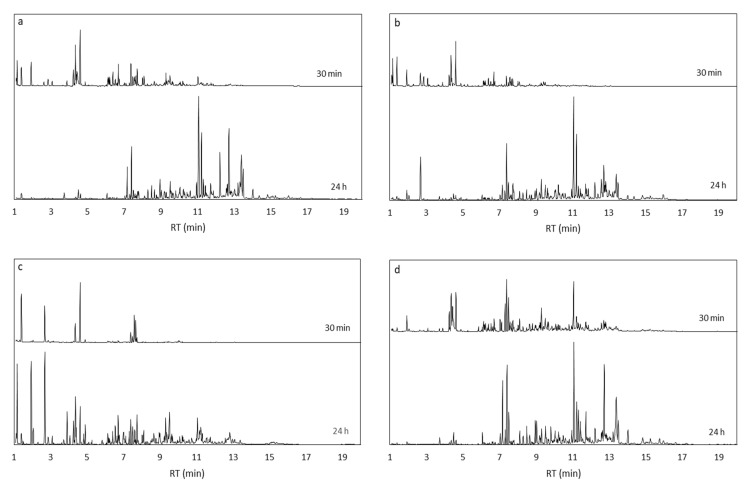
Chromatograms obtained from the analysis by GC-MS of derivatized reaction products at 30 min and 24 h for (**a**) ZIF-67, (**b**) ZIF-67 exchanged 1:1, (**c**) ZIF-67 exchanged 1:2, and (**d**) ZIF-8.

The chromatograms obtained from the analysis of the derivatized products for FR carried out with each MOF are shown in Figure 5. As expected, numerous peaks are observed, indicating the formation of a large number of different products. The exact identification of each product in a typical formose mixture is quite challenging, and quantifying the reaction products in this complex matrix is often impossible [64,65]. However, despite the complexity, we were able to distinguish monosaccharides with different chain lengths and identify polyols, aldoses, and ketoses, which are typical products coming from FR, which clearly indicates the real possibility of using this kind of material for promoting this reaction. The products are well separated as a function of the chain length. Thus, we can find C_3_ monosaccharides, such as glyceraldehyde and dihydroxyacetone, between 3 and 5 min. Between 6 and 9 min, we find C_4_ sugars, for example, erythrose and erythrulose; at retention times from 9 to 11, C_5_ products such as ribose can be found, while C_6_ fructose and glucose appear after 11 min. The large peaks before minute 3 are unreacted glycolaldehyde, residues from the derivatization procedure, or subproducts (see Supplementary Material).

Comparing the chromatograms obtained at 30 min, we observed that mainly short-chain linear monosaccharides are formed, with C_3_ and C_4_ sugars being the main products, whereas, once the reaction proceeds for 24 h, the formose mixture becomes more complex, and we observed longer carbon chains and a variety of different products, such as ribose, ribulose, arabinose, xylitol, fructose, tagatose, glucitol, and dulcitol. The number of products obtained made the identification process quite complex at this point; several isomers were found for C_5_ and C_6_ sugars (ketoses, aldoses together with furanose, and pyranose forms of the same monosaccharide), as well as polyols, and that led to overlapping among peaks.

Comparing the chromatograms obtained at 30 min, it is noteworthy that ZIF-67 exchanged 1:2 seems to moderate the number of products obtained as compared with other catalysts at the same reaction time. In this case, only glyceraldehyde, threose (C_4_ aldose, stereoisomer of erythrose), and erythrulose (C_4_ ketose, structural isomer of erythrose) were observed. The reason for this increased selectivity is probably related to the difference in FA conversion. In fact, the conversion of FA at 30 min of reaction time by using ZIF-67 exchanged 1:2 is only 30%, while it is around 70% with all other MOFs. Low conversions with these catalysts may help keep the formose mixture simple, limiting the products obtained to some short-chain monosaccharides. At higher FA conversion, longer-chain monosaccharides and also polyols were observed. It is interesting to observe how, under the same conditions, ZIF-67 exchanged 1:2 makes the reaction less active, which can result in an important advantage since the uncontrolled reactivity of the FR is its main drawback [3,4].

**Figure 6:**
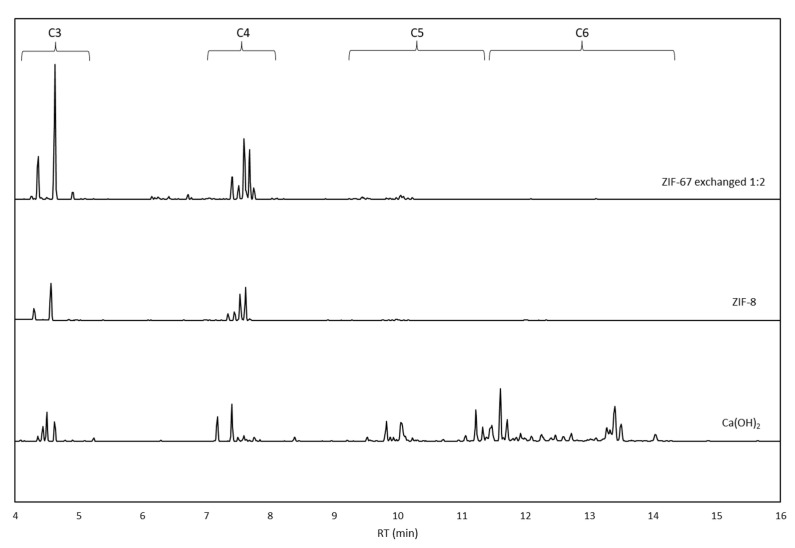
Chromatograms obtained from FR at 30% conversion for MOFs compared to a traditional catalyst. From top to bottom: ZIF-67 exchanged 1:2, ZIF-8, Ca(OH)_2_. The retention times associated with different chain lengths are highlighted for better understanding.

The favored formation of short-chain linear monosaccharides at low FA conversion was confirmed by the analysis of products obtained using the commercial ZIF-8 as catalyst after 1 min of reaction time (where the FA conversion obtained was around 30%). As shown in Figure 6, the products obtained with ZIF-8 are also limited, which supports the relationship between the selectivity and the rate of conversion when MOFs are used as catalysts. Further, the performance of the presented MOFs (i.e., ZIF-67 exchanged 1:2 and ZIF-8) differs from what was observed with the most common catalyst reported in the literature, Ca(OH)_2_. If compared with the classical formose catalyst Ca(OH)_2_, for similar FA conversions (around 30%), both MOFs show enhanced selectivity with cleaner chromatograms containing only C_3_ (glyceraldehyde) and C_4_ (threose and erythrulose) species, with traces of C_5_ products (Table 1). On the contrary, even at low conversions, the chromatogram obtained with Ca(OH)_2_ shows a significantly more complex mixture containing linear monosaccharides and polyols with chain lengths ranging from C_3_ to C_6_ (Table 1). That clearly indicates the important role that MOFs could play in addressing the challenging task of controlling the selectivity during the FR.

The reason for the difference between MOFs and Ca(OH)_2_ can be related to the intrinsic characteristics of these catalysts. While the exact formose mechanism of the reaction is still under debate, it is clear that Ca(OH)_2_ acts as a homogeneous catalyst, with the CaOH^+^ in solution recognized as the actual active species that catalyzes the reaction. Moreover, Ca^2+^ in solution is capable of coordinating the enolates formed as intermediates of the reaction and facilitating the aldol condensation that leads to monosaccharides of higher size [6,13]. Even if some examples of increased selectivity with Ca(OH)_2_ have been reported in the past [7], the reality is that controlling the FR with this homogeneous catalyst is quite challenging. In fact, as soon as the first short-chained monosaccharides are formed in the formose mixture, if they are able to form enolates, the reaction proceeds quickly, forming longer products by the continuous addition of FA to the chains. In addition, Ca(OH)_2_ is a strong base, and it is known to efficiently catalyze the Cannizzaro disproportion [7,10,13], a side reaction that can compete with the FR mechanism, which would increase the number of different products obtained. With no limitation in structural porosity and a strong unselective basicity, Ca(OH)_2_ is not able to foster the selectivity of the reaction, and the formation of the products is usually fast and disordered.

The increased selectivity observed with MOFs may be connected to the nature of heterogeneous catalysts (which will be discussed later on) and to a combination of their structure and basicity. A certain Lewis basicity originates from the coordination Me-N [66], but the presence of defects in the framework structure is also an important source of acid–base sites in both ZIF-67 and ZIF-8, as described by Naz et al. In their work, the amount of basic sites in ZIF-67 and ZIF-8, evaluated as a result of a CO_2_ TPD characterization, was reported to be 5.25 µmol/g and 4.99 µmol/g, respectively [67]. The framework of ZIF-67 and ZIF-8 is sodalite type and presents pores of 11.6 Å connected through eight six-membered ring windows of 3.4 Å and six four-membered ring small pores of 0.8 Å [68,69,70]. The substituted ZIF-67 presents the same crystalline structure except for a contraction observed from the XRD pattern, which indicates a reduction in the d-spacing due to the exchanged linkers. This effect is more pronounced for ZIF-67 exchanged 1:2, which exhibits the most contracted structure. It is likely that small FA molecules, with an effective size of 2.5 Å [71], are able to enter into the large and medium pores of the ZIFs, but the reaction and formation of large monosaccharides inside the structure may be unfavored due to the limited space. Considering, for example, that a glucose molecule has a size of 8.6 Å and 8.4 Å measured on the long and short axes of its ring form and 15 Å in its open-chain form (BNID 110,368 and 106,979 [72]), the formation of such monosaccharides is most likely to occur on the outer surface of the catalyst. On the other hand, the inner surface of the ZIF pores, which is easily accessible only for small molecules, may be responsible for the formation of short chain monosaccharides at low conversions. Further, the contraction in the ZIF structure observed when the 2-methylimidazole is substituted with 1,2,4-1H-triazole may be the reason for the slower reaction observed with ZIF-67 exchanged 1:2. In fact, ZIF-67 exchanged 1:2 exhibits the most significant contraction, as can be observed in the XRD patterns (Figure 1), which supports that molecules spend more time to diffuse into the pores, resulting in a slower, and easy to control, reaction by using this MOF as catalyst. This effect is not observed with ZIF-67 exchanged 1:1, which exhibits a similar reactivity than the rest of the MOFs, because the content of 1,2,4-1H-triazole is less in this catalyst, and its structure contraction is less dramatic. This shape selectivity given by product diffusion is one of the advantages of using porous solids as catalysts for liquid phase reactions [73]. The metals in ZIF-67 and ZIF-8 are coordinated with four nitrogen atoms each [74]; therefore, the rings of the basic ligands 2-methylimidazole and 1,2,4-1H-triazole are blocked in a fixed geometry. This may benefit the reaction path by slowing the reaction and inhibiting competitive side mechanisms.

Evidently, MOFs are solid materials, so it is logical to think that the reaction is controlled heterogeneously. However, since the ligands of the MOFs could be detached to some degree, leading to homogeneous catalysis, we explored the migration of the linkers from the MOF to the solution. Thus, in order to determine if the linkers leach from the solid and if the contribution of such organic bases in solution could play a significant role in the catalytic activity and selectivity of the FR, we carried out an experiment removing the solid catalyst after 1 min of reaction, in a similar procedure to that described by Nguyen et al. [62]. After removing the solid catalyst, the reaction was kept under continuous stirring and temperature for 24 h. If the reaction continued after removing the solid, it would mean that the linkers in solution were the actual catalyst. Products were analyzed after 24 h, and, indeed, no reaction was observed, indicating that the FR can take place only if solid MOFs are present.

Moreover, to further demonstrate that it is effectively the solid which catalyzes the reaction, we determined the amount of leached metal in the solution after 30 min by removing the solids and analyzing the solution for metals with ICP. The results are summarized in Table 2. The amount of metal dissolved is calculated based on the initial concentration of MOFs.

As indicated by the results of the ICP analysis, no significant leaching occurs at the reaction conditions. Nevertheless, we performed an additional experiment to further prove that no contribution from the linkers or metal in solution is given to the FR catalysis. Based on the ICP results, we carried out an FR using only the precursors of the MOFs in the concentration that they were found in the solution. No reaction occurred when Co (NO_3_)_2_, 2-methylimidazole, or 1,2,4-1H-triazole were used, further confirming that the solid MOFs are indeed the actual catalysts of the FR (i.e., FR is promoted by means of heterogeneous catalysis).

One of the main advantages of heterogeneous catalysts is the ease of separation as compared to homogeneous ones, which allows the reuse of the material. To verify the catalysts’ stability, MOFs were recovered after the first 24 h, centrifuged at 4000 pm, washed three times with ethanol and water, and dried in an oven at 80 °C. The stability tests were carried out after 24 h of reaction with the aim to have similar conversion values (in the range of 60–80%) and product distribution for the four catalysts under study. They were then reused for a 24 h FR to evaluate the reproducibility of the results. As shown in Table 3, the conversion at 24 h reaction time was found to be quite reproducible. All the ZIF-67 showed a slight reduction in the catalytic activity, while ZIF-8 increased its catalytic activity in this second cycle, although this effect could be explained by the evaporation of the FA in solution, as the reaction was carried out in a little volume.

**Figure 7:**
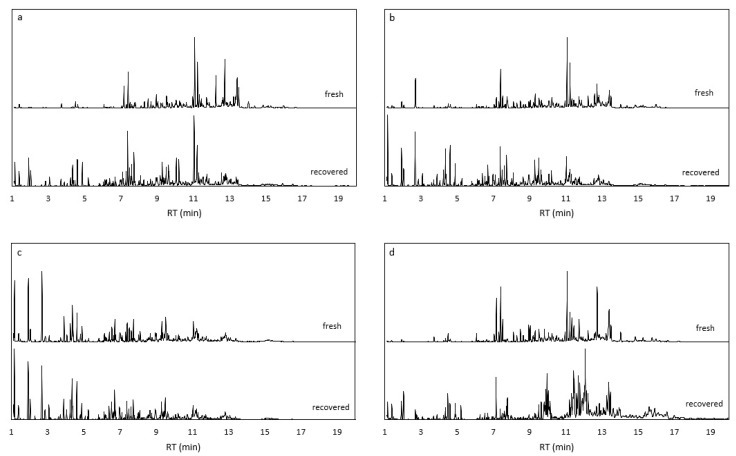
Chromatogram comparison of derivatized products obtained from an FR carried out at 60 °C with fresh (top) and recovered (bottom) MOFs at 24 h reaction time. (**a**) ZIF-67, (**b**) ZIF-67 exchanged 1:1, (**c**) ZIF-67 exchanged 1:2, (**d**) ZIF-8.

**Figure 8:**
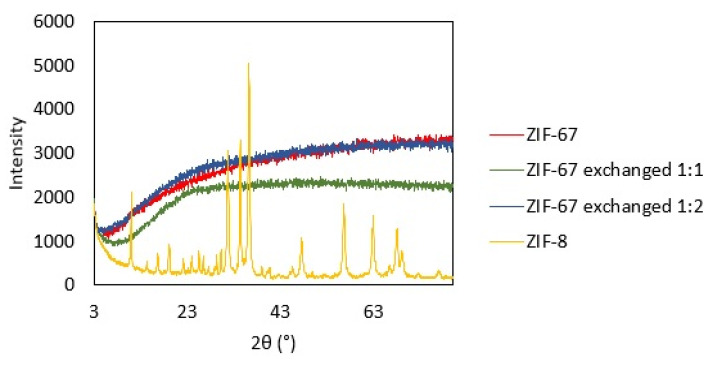
XRD patterns of MOF catalysts recovered by centrifugation and washed with water and ethanol, after 24 h reaction at 60 °C.

The products of the second FR with the recovered MOFs were also analyzed. Figure 7 compares the chromatograms obtained from fresh and recovered catalysts after 24 h of reaction time. All the MOFs recovered are still active for the FR reaction. The distribution of the products shows a typical formose mixture, consisting of a variety of C_3_ to C_6_ linear monosaccharides and polyols. The reproducibility of the products obtained before and after the recovery seems only to be almost identical in the case of the ZIF-67 exchanged 1:2, while the rest of the catalysts show different product patterns comparing the fresh and recovered MOF. We characterized the recovered MOFs by XRD to investigate the stability of the crystalline phases of the materials after one reaction cycle (Figure 8).

As can be observed, the original pattern is not conserved, indicating that the original framework of the materials is compromised. ZIF-67 and its substituted versions show, after the recovery, an amorphous pattern, while ZIF-8 presents some peaks, but they are attributable to a different phase than sodalite.

Structural instability is a typical drawback when working with MOF materials. In this application, the temperature applied for the reaction is quite below the limit for MOF stability (i.e., above 100 °C for substituted ZIF-67, and up to 300 °C for ZIF-67 and ZIF-8 [75,76]); however, the high concentration of organic species in the medium may be the cause of a weakening of the metal–ligand bonds, which finally results in a reassessment of the structure and the loss of the crystallinity. The fact that catalysts do not lose their catalytic activity, even if their framework is compromised, is explained by the fact that the active sites responsible for the catalysis are still present, even if they are not in a crystalline form. The FR can still take place; however, when the structural integrity of the material is lost and the framework collapses, the inner surface is not accessible anymore, and the outer surface is the only one exposed. Therefore, there is no limitation in product formation as a result of the porous structure.

## 3. Materials and Methods

ZIF-67 is obtained directly from its precursors. A solution of 2-methylimidazole and Co(NO_3_)_2_ in the same solvent are mixed at room temperature, shaken for 2 min, and left to stand for some time for crystallization. A purple product is obtained, which is separated by centrifugation and washed twice with ethanol. The solid is then dried in an oven at 60 °C overnight.

The ligand exchange is performed by mixing a methanolic suspension of ZIF-67 with a solution of 1,2,4-1H-triazole in methanol. The resulting mixture is then stirred for a duration of 3 days. Afterwards, the MOF is separated through centrifugation and subjected to 10 washes using methanol. This process is carried out to remove any ligands that are not firmly attached to the structure. Different concentrations of triazole were utilized in the exchange process to prepare two different substituted ZIF-67, with a methylimidazole/triazole proportion of 1:1 and 1:2.

Crystallographic phases were identified by powder X-ray diffraction (PXRD) recorded on Panalytical Empyrean multifunctional equipment (Malvern Panalytical Ltd., Malvern, UK) for X-ray diffraction analysis, which, in its basic configuration, has a goniometer with an X-ray tube with a Cu Kα cathode and a Ni filter. It was operated in an angular scanning range of 3 to 40° at an angular velocity of 1°/min and at room temperature. To carry out the FRs, the conditions were optimized based on the type of material used to catalyze the reaction to ensure a fair comparison. The procedure followed when MOFs are used as catalysts: start transferring 30 µL of FA 37% wt. into a 2 mL microtube. Then, 0.03 g of GA and 1 mL of deionized water is added, and the tube is placed in a rocking shaker, maintaining the temperature at 60 °C. As soon as the temperature reaches a stable value, 20 mg of the corresponding MOF under consideration is incorporated to the mixture. The reaction is allowed to proceed for 24 h at 300 rpm.

The porosity of the samples was characterized by means of nitrogen adsorption–desorption isotherms. Samples were outgassed at 150 °C for 4 h prior to the adsorption measurements. The nitrogen adsorption–desorption isotherms were measured at −196 °C in a Quadrawin (Quantachrome, Graz, Austria) device. SBET was determined using more than 5 points in the BET equation, and the value of c was always positive. The pore size distribution was obtained by the Horvath–Kawazoe differential pore volume method.

TGA was carried out in a TGA/SDTA851e/LF/1600 apparatus from Mettler Toledo under a dynamic atmosphere of Ar (100 cm^3^/min), with a heating rate of 10 °C/min.

The performance of the reaction is determined at different times to study the evolution of the FA concentration (activity) and to identify the products obtained (selectivity). Hence, sampling is carried out at a reaction time of zero, prior to the addition of the MOF, at 30 min, and, finally, at 24 h from the addition of the catalyst.

On the other hand, the FR analysis by using Ca(OH)_2_ as catalyst is carried out as follows: 100 mL of FA solution 1 M, 0.2 g of GA, and 1 g Ca(OH)_2_ are added into a three-necked round bottom flask and placed in a heating mantle with temperature control and magnetic stirring. The temperature is maintained at 50 °C at a constant stirring until complete conversion of the FA. As the rate of the reaction is very fast once Ca(OH)_2_ acts as catalyst, several samples have to be taken in order to properly study the FA consumption and product formation. Hence, the sampling time is fixed at 10 s, 30 s, 1 min, 2 min, 5 min, and 10 min.

For FA quantification, an aliquot of 100 µL is taken and derivatized with 2,4-dinitrophenylhydrazine to obtain the correspondent 2,4-dinitrophenylhydrazone, and it is analyzed by HPLC with an Agilent 1260 Infinity equipped with a DAD detector (Agilent, Santa Clara, CA, USA) and a LiChrospher^®^ 100 RP-18 endcapped column, purchased from Merck (Darmstadt, Germany). For products identification, an aliquot of 200 µL is taken and immediately frozen in liquid nitrogen. The frozen sample is lyophilized, and the dried product is redissolved in pyridine, derivatized in two steps with O-Ethylhydroxylamine hydrochloride and N,O-Bis(trimethylsilyl)trifluoroacetamide, according to the procedure described by Haas et al. [64], and analyzed with an Agilent 8890 gas chromatograph (Agilent, Santa Clara, CA, USA) equipped with a mass detector. The separation was performed in a CP-Sil 8 CB column purchased from Agilent Technologies (Agilent, Santa Clara, CA, USA).

Four MOFs were tested as catalysts for FR: ZIF-67, ZIF-67 exchanged 1:1, ZIF-67 exchanged 1:2, and ZIF-8. The ZIF-67 series were synthesized in-house, while ZIF-8 was purchased from Merck. The ZIF-8 differs from ZIF-67 only for the metal contained in the framework (Zn instead of Co). In order to evaluate the stability of the materials, all the catalysts tested were recovered and reused. For this purpose, once the FR reached 24 h, the reaction suspension was centrifuged with a Nuve NF400 (Nuve, Ankara, Türkiye) centrifuge at 4000 rpm to separate the catalyst from the solution. The solid so obtained was washed three times with deionized water and ethanol 99.8 vol.%, centrifuged, and then dried in an oven at 80 °C overnight. The catalysts recovered following this procedure were reused for FR at the same conditions described above in order to test the reproducibility of the catalytic activity. Thus, FA conversion and product distribution were studied after 24 h of reaction time and compared with the results obtained with fresh catalysts.

The comparison of fresh and used materials was also supported by the characterization of fresh and recovered catalysts by X-ray diffraction (XRD).

To demonstrate the heterogeneous catalysis, and to exclude the contribution of homogeneous ones, an FR was carried out where the MOFs were removed from the solution through centrifugation after 1 min of reaction at 60 °C. The remaining solution was left at 60 °C for 24 h, and, after that, the FA conversion and product distribution was analyzed. Furthermore, the catalysts were tested to check whether, at the reaction condition, they presented leaching of the metal from the network to the solution. For this purpose, an FR reaction was carried out for 30 min; after that, the solid was separated by centrifugation, and the remaining solution was analyzed with inductively coupled plasma (ICP) to detect the presence of Co for ZIF-67 and Zn for ZIF-8. The actual metal in the reaction mixture was determined in a PerkinElmer device (Optimal 3000, Perkin Elmer, Waltham, MA, USA). To further exclude the contribution of homogeneous catalysis given by the precursors of the MOFs that could exist in solution at the reaction conditions, FR was carried out using only the precursors of the MOFs: 2-methylimidazole, 1,2,4-1H-triazole, and Co(NO_3_)_2_. The amount of MOF precursor to be used for these experiments was calculated based on the results of the ICP. The reaction mixture was analyzed for FA conversion and product distribution at 30 min and 24 h reaction time.

## 4. Conclusions

In this paper, the use of MOFs as heterogeneous catalysts for the FR is explored for the first time. Four MOFs were used as catalysts for the FR: a synthesized ZIF-67, two substituted ZIF-67 obtained from it, and a commercial ZIF-8. The materials were characterized by XRD, and their catalytic performances for the oligomerization of FA were evaluated. All the materials showed remarkable catalytic activity for the FR, reaching FA conversion up to 80% after 24 h in the first cycle. An increased selectivity towards small linear monosaccharides was found, which was ascribed to a combination of the porous structures and the mild basic properties of the MOFs. Heterogeneous catalysis was confirmed with two experiments that exclude the contribution of leaked organic linkers from the solid to the catalytic activity. The catalysts were recovered and reused for a second cycle of FR to evaluate the stability of the materials. In the second cycle of the reaction, the catalytic activity was maintained, but the materials suffered from degradation of their crystalline framework, apparently caused by the high concentration of organic species present in the medium. Future works should address the loss of the MOF integrity under the reaction conditions, exploring the possibility of using new families of MOF materials with higher robustness. Different linkers should be considered for being introduced to the MOFs to preserve the structural stability if a high FA conversion is required and the materials need to be recovered and reused. Nevertheless, this work opens the path for new research to further elucidate the mechanism that leads to enhanced selectivity in the FR and to obtain MOFs with increased stability that could be suitable to better resist FR conditions for several cycles.

## Figures and Tables

**Table 1:** Selectivity to C_2_–C_6_ species for ZIF-67 exchanged 1:2, ZIF-8, and Ca(OH)_2_ at 30% conversion.

Catalyst	C_3_	C_4_	C_5_	C_6_
Ca(OH)_2_	12	15	10	63
ZIF-67 exchanged 1:2	50	47	3	--
ZIF-8	41	58	1	--

**Table 2:** ICP results for leaching analysis of MOF solution samples, obtained by removing the solid by centrifugation after 30 min of reaction at 60 °C.

Catalyst	Metal	Conc. (ppm)	Amount of Metal Dissolved (%)
ZIF-67	Co	11.5	0.3
ZIF-67 exchanged 1:1	Co	8.4	0.2
ZIF-67 exchanged 1:2	Co	13.3	0.3
ZIF-8	Zn	25.6	0.5

**Table 3:** FA conversion comparison for fresh catalysts and catalysts recovered by centrifugation after 24 h of reaction.

Catalyst	Fresh	Recovered
ZIF-67	Conversion 76%	Conversion 70%
ZIF-67 exchanged 1:1	Conversion 76%	Conversion 61%
ZIF-67 exchanged 1:2	Conversion 60%	Conversion 49%
ZIF-8	Conversion 81%	Conversion 75%

## Data Availability

Data is unavailable due to privacy restrictions.

## Supplementary Materials

The following supporting information can be downloaded at: https://www.mdpi.com/article/10.3390/molecules28166095/s1.
