# Supplementary material for: Metal–Organic Frameworks as Formose Reaction Catalysts with Enhanced Selectivity
Source: Molecules. 2023 Aug 17;28(16):6095. doi: 10.3390/molecules28166095 (PMC10458508; doi:10.3390/molecules28166095)
Supplement: Supplementary file 1 [file molecules-28-06095-s001.zip › molecules-2530752-supplementary.pdf]

Supporting Information

List of figures

Figure S1: Reaction and analysis setup that permits the quantification of FA and identification of formose products. 2

Figure S2: HPLC calibration curve of DNPH-derivatized FA for quantification 2

Figure S3: Chromatogram obtained from HPLC quantification of FA. The peak of the excess of DNPH is visible and completely separated from that of FA. 3

Figure S4: Mass spectrum of peak at RT 1.164 min, identified as N-trimethylsilyloxymethyl trifluoroacetamide 3

Figure S5: Mass spectrum of peak at RT 1.394 min, identified as the TMS derivative of N-Formylglycine. 3

Figure S6: Mass spectrum of peak at RT 1.405 min, identified as the TMS derivative of 1,4-Dioxane-2,3-diol (glycolaldehyde dimer) 4

Figure S7: Mass spectrum of peak at RT 1.919 min, identified as the 2TMS derivative of ethylene glycol. 4

Figure S8: Mass spectrum of peak at RT 2.686 min, identified as the TMS derivative of pipecolic acid. 4

Figure S9: Mass spectrum of peak at RT 2.850 min, identified as (butoxymethyl)trimethylsilane 5

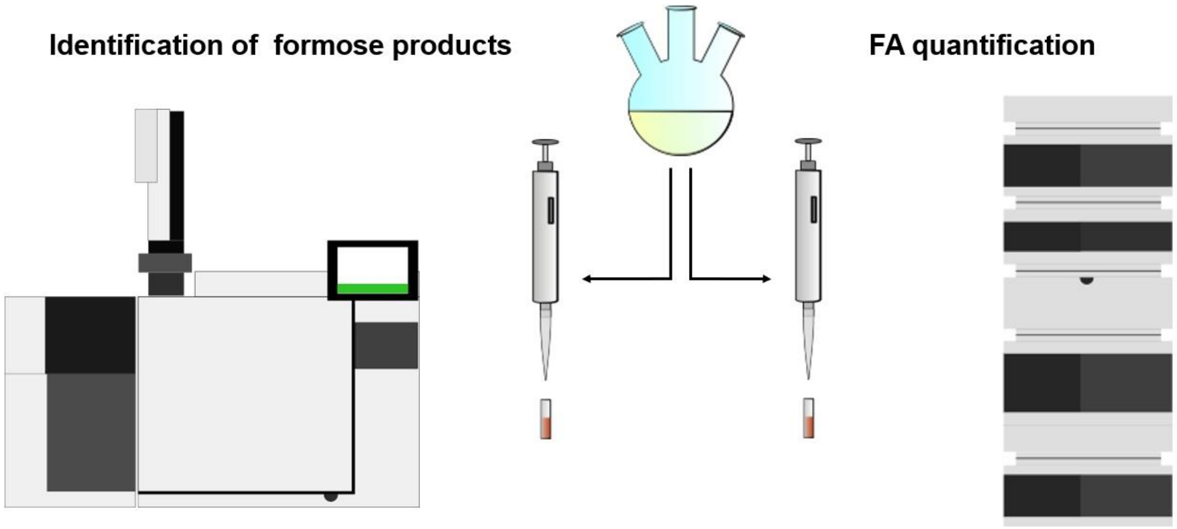

Figure S1: Reaction and analysis setup that permits the quantification of FA and identification of formose products.

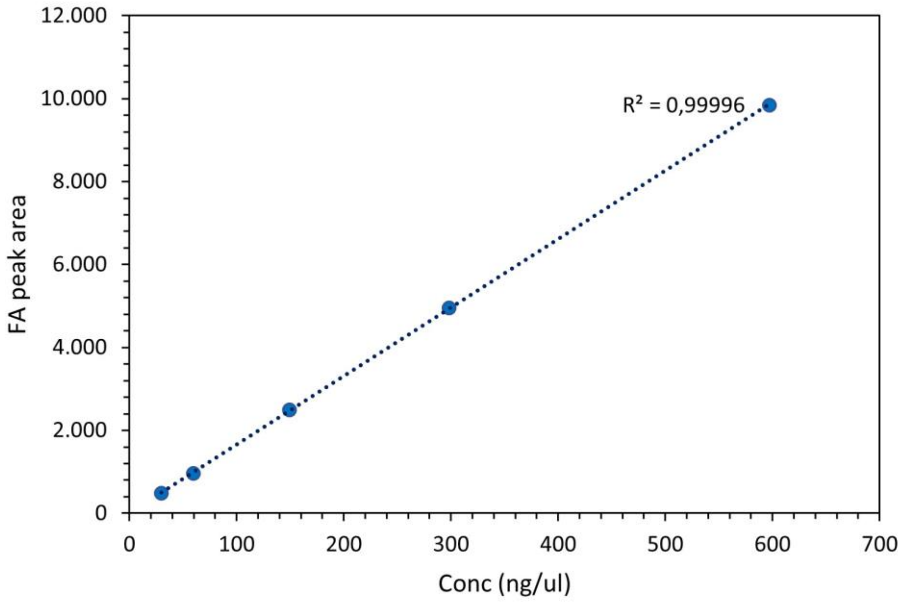

Figure S2: HPLC calibration curve of DNPH-derivatized FA for quantification

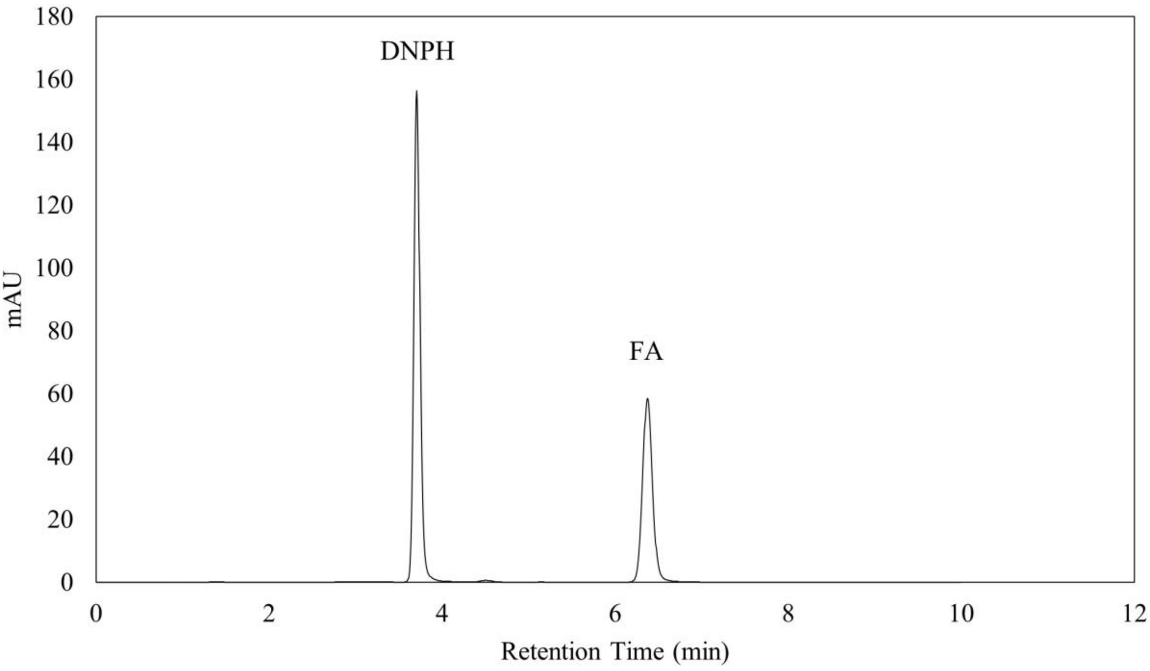

Figure S3: Chromatogram obtained from HPLC quantification of FA. The peak of the excess of DNPH is visible and completely separated from that of FA.

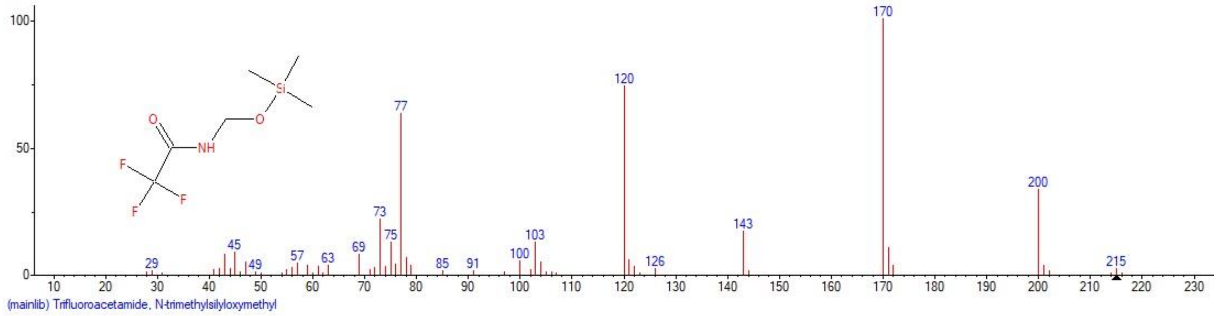

Figure S4: Mass spectrum of peak at RT 1.164 min, identified as N-trimethylsilyloxymethyl trifluoroacetamide

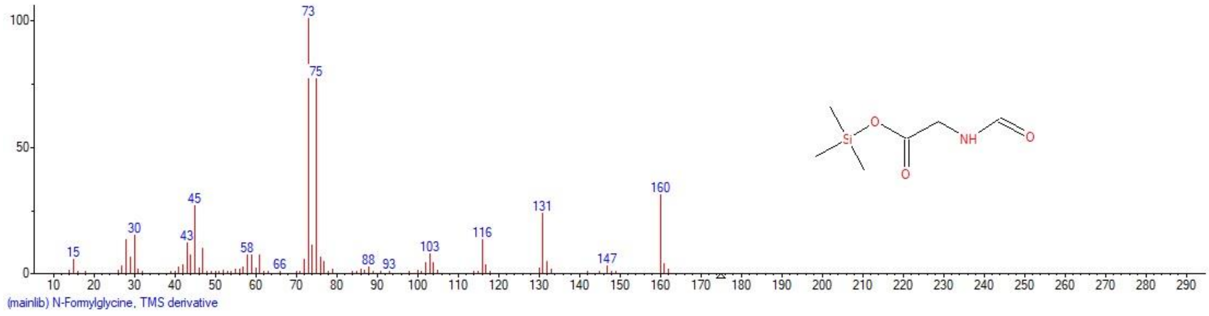

Figure S5: Mass spectrum of peak at RT 1.394 min, identified as the TMS derivative of N-Formylglycine.

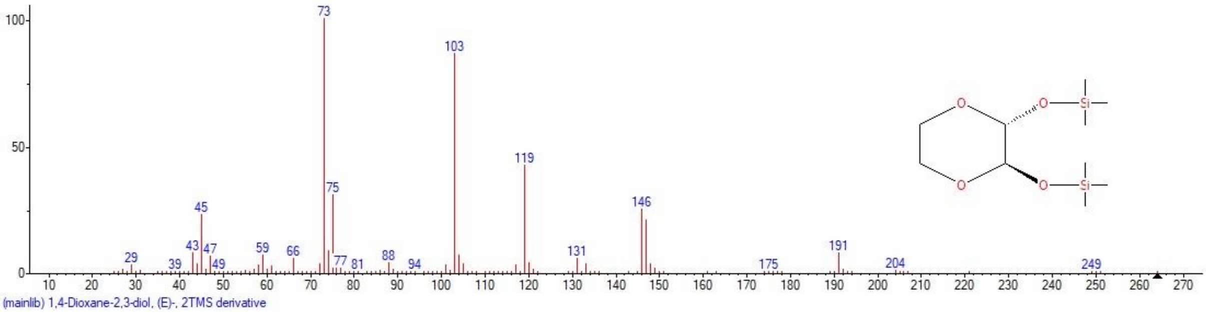

Figure S6: Mass spectrum of peak at RT 1.405 min, identified as the TMS derivative of 1,4-Dioxane-2,3-diol (glycolaldehyde dimer)

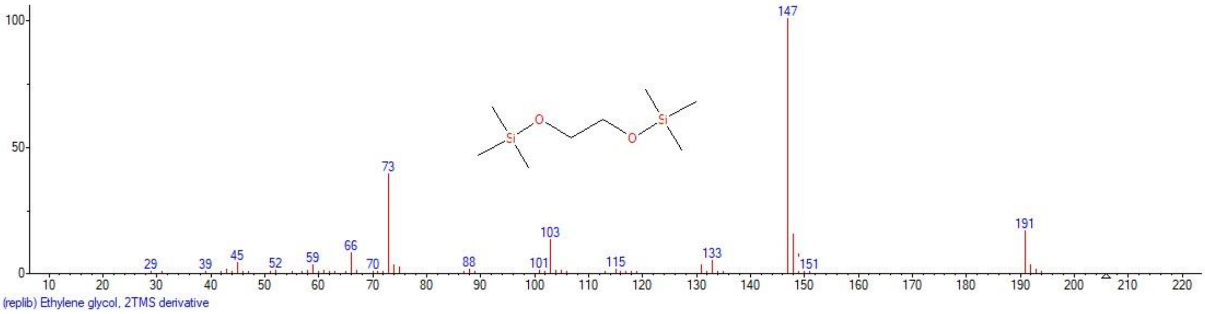

Figure S7: Mass spectrum of peak at RT 1.919 min, identified as the 2TMS derivative of ethylene glycol.

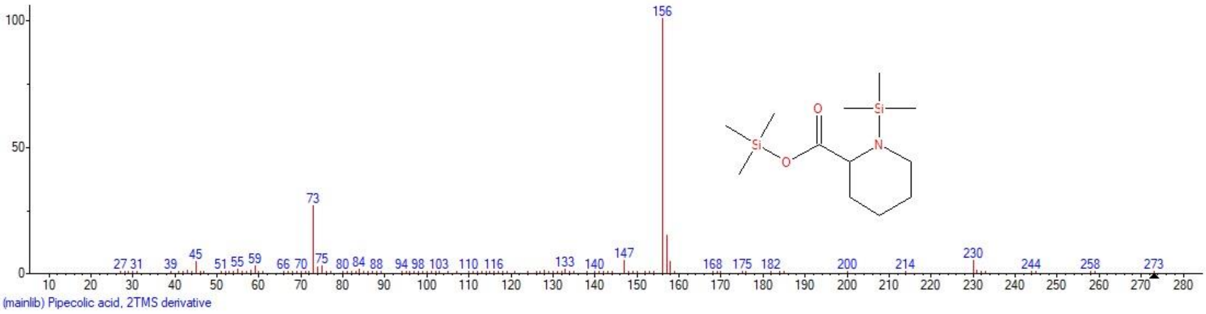

Figure S8: Mass spectrum of peak at RT 2.686 min, identified as the TMS derivative of pipecolic acid.

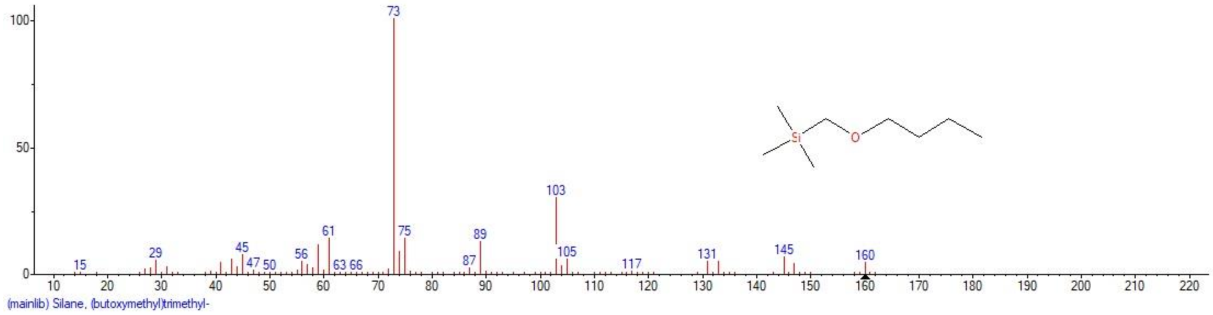

Figure S9: Mass spectrum of peak at RT 2.850 min, identified as (butoxymethyl)trimethylsilane
